# A Case of Severe Somatized Depression in a Young Adult: Diagnostic Challenges

**DOI:** 10.1192/j.eurpsy.2023.1627

**Published:** 2023-07-19

**Authors:** O. M. Pityk, I. M. Kuzhda, M. I. Pityk

**Affiliations:** 1 Psychiatry, Narcology and Medical Psychology, Ivano-Frankivsk National Medical University; 2 Ophtalmology, Ivano-Frankivsk Regional Children Hospital; 3 Neurology, Ivano-Frankivsk National Medical University, Ivano-Frankivsk, Ukraine

## Abstract

**Introduction:**

Depressive disorders in adolescence and young adulthood have always been and remain an urgent problem due to their fairly high prevalence among the population, serious difficulties in diagnosis and untimely treatment. Timely diagnosis and adequate treatment can have a powerful impact on the future life of a person in a positive context. This process requires both standardized mechanisms, and an individual detailed study of each case, as the future of the individual depends on it.

**Objectives:**

A young adult M., 20 years old, a university student, from a socially prosperous family, approached us.

Main complaints: headaches that have been going on for almost 4 years. Pains did not depend on loads, both physical and mental, were of various characteristic and different localization. Non-steroidal anti-inflammatory drugs, as well as anti-migraine drugs, have little effect on the pathological experiences.

**Methods:**

Our main method of examination was clinical interview. In the complex assessment detailed neurological and ophtalmological examination included.

**Results:**

The parents referred patient for a medical examination about a year ago, because they noticed persistent mydriasis in him. During the year, the patient underwent a detailed examination (consultations of a therapist, endocrinologist, neurologist, ophthalmologist, MRI, EEG, dopplerography). Doctors expressed various assumptions about the diagnosis, because all the studies did not reveal any pathology that could explain the indicated complaints and mydriasis.

During the initial interview, a high level of intelligence and knowledge was revealed, as well as a sufficient ability to learn. Examining the emotional-volitional sphere, a slight level of emotional instability, mild irritability, anhedonia and a slight degree of hypobulia (which can be explained by long-lasting and persistent pathological somatic experiences in the form of headaches) were found. Incomplete Protopopov’s triad was revealed.

The patient was referred for repeated neurological and ophthalmological examination. Specialists with a high qualification level discovered the A. Athanassio symptom in him.

He was diagnosed with recurrent depressive disorder, a current episode of severe depression with somatic symptoms, and appropriate treatment was prescribed.

**Conclusions:**

1. Depressive disorders in adolescence and young adulthood require special attention from specialists of all medical specialties.
2. The need for a detailed medical examination and modern neuroimaging methods is beyond doubt.
3. Psychiatric examination cannot be limited to assessment of mental status only, and assessment of Protopopov’s triad should be part of psychiatric examination.
4. Neurological and ophthalmological examination must necessarily include an assessment of neuro-ophthalmological symptoms.
5. Individual selection of treatment should be carried out by a psychiatrist.

**Sorry to say, Professor Mykola Pityk died 27.12.2020**

**Disclosure of Interest:**

None Declared

